# Highly Sensitive and Selective Toluene Gas Sensors Based on ZnO Nanoflowers Decorated with Bimetallic AuPt

**DOI:** 10.3390/molecules29071657

**Published:** 2024-04-07

**Authors:** Huiting Peng, Yiping Liu, Yinfeng Shen, Ling Xu, Jicun Lu, Ming Li, Hong-Liang Lu, Liming Gao

**Affiliations:** 1 State Key Laboratory of Metal Matrix Composites, School of Material Science and Engineering, Shanghai Jiao Tong University, Shanghai 200240, China; pddbeibei0512@sjtu.edu.cn (H.P.); liuyp37@sjtu.edu.cn (Y.L.); syf1999@sjtu.edu.cn (Y.S.); mingli90@sjtu.edu.cn (M.L.); 2 Guanghua Lingang Engineering Application Technology Research and Development (Shanghai) Co., Ltd., Shanghai 201306, China; lingxu@fudan.edu.cn (L.X.); jerrylu126@126.com (J.L.); 3 State Key Laboratory of ASIC and System, Shanghai Institute of Intelligent Electronics & Systems, School of Microelectronics, Fudan University, Shanghai 200433, China

**Keywords:** ZnO nanoflowers, AuPt alloy, gas sensor, toluene detection

## Abstract

Efficient sensors for toluene detecting are urgently needed to meet people’s growing demands for both environment and personal health. Metal oxide semiconductor (MOS)-based sensors have become brilliant candidates for the detection of toluene because of their superior performance over gas sensing. However, gas sensors based on pure MOS have certain limitations in selectivity, operating temperature, and long-term stability, which hinders their further practical applications. Noble metals (including Ag, Au, Pt, Pd, etc.) have the ability to enhance the performance of MOS-based sensors via surface functionalization. Herein, ZnO nanoflowers (ZNFs) modified with bimetallic AuPt are prepared for toluene detection through hydrothermal method. The response of a AuPt@ZNF-based gas sensor can reach 69.7 at 175 °C, which is 30 times, 9 times, and 10 times higher than that of the original ZNFs, Au@ZNFs, and Pt@ZNFs, respectively. Furthermore, the sensor also has a lower optimal operating temperature (175 °C), good stability (94% of previous response after one month), and high selectivity towards toluene, which is the result of the combined influence of the electronic and chemical sensitization of noble metals, as well as the unique synergistic effect of the AuPt alloy. In summary, AuPt@ZNF-based sensors can be further applied in toluene detection in practical applications.

## 1. Introduction

An increase in the concentration of volatile organic compounds (VOCs) has been observed in the atmosphere year by year with the continuous expansion of global industry [1]. A large amount of VOCs, especially benzene, toluene, ethylbenzene, and xylene (BTEX), existing in the atmosphere will cause serious negative impacts on both human health and the environment [2,3,4,5]. Toluene(C_6_H_5_CH_3_) is a typical aromatic compound widely used in furniture, paint, pesticides, leather, laboratory solvents, inks, and automotive fuels [6,7,8,9]. However, long-term exposure to toluene can cause various adverse effects to the human skin, kidneys, heart, respiratory system, nervous system, etc. [10,11,12]. It has been proven that toluene can cause a variety of diseases, such as asthma, nasopharyngeal cancer, etc., and is listed as the 3rd most dangerous carcinogen by the WHO [13]. Standards from the UK Health Protection Agency indicate that the human body can only stay in the environment with a toluene content of 50 ppm for a maximum of 8 h, or it will cause serious adverse reactions and even threaten life safety [14]. Therefore, the accurate detection of toluene at low temperatures and concentrations is essential.

At present, there are many instruments and technologies used for detecting toluene, including gas chromatography–mass spectrometry, fluorescence spectroscopy, FTIR analysis, etc. [1]. Among them, chemical resistive metal oxide semiconductor (MOS) gas sensors have become one of the most attractive sensors in recent years, due to their unique advantages in manufacturing costs, response speeds, selectivity, and sensitivity. Wherein, both p-type MOS-based sensors (NiO [15], CuO [16], etc.) and n-type MOS-based sensors (ZnO [11], SnO_2_ [17], etc.) are widely applied in toluene detection. Generally, n-type MOS-based exhibits superior sensitivity over p-type MOS-based gas sensors. Up to now, ZnO is a promising material for gas sensing with wide band gap (3.37 eV) [18], high charge mobility (~200 cm^2^/V·s), large exciton binding energy (60 meV) [19], and good chemical stability and thermal stability. However, gas sensors based on bulk materials of metal oxide exhibit unsatisfactory sensing performance, such as low sensitivity, poor selectivity, and high power consumption, due to their low specific surface area [20]. Compared to bulk materials, ZnO nanomaterials exhibit better catalytic properties due to surface and interface effects, small size effects, etc. To improve, nanostructures with high specific sensing areas, such as nanowires [21], nanorods [22], nanofibers [23], nanoflakes [24], nanospheres [25], and nanoflowers [26], have been successfully reported to achieve better sensing performance as more active sites are provided for the adsorption of target gases [27]. The flower-like ZnO structure promotes efficient adsorption of target gases due to its branched structure and large specific surface area, leading to a positive impact on the working temperature, sensitivity, and response speed. For example, a flower-like ZnO gas sensor is reported with a response of 31.81 for 50 ppm toluene, as well as the ability to still work at a low temperature (20 °C) with a certain response (1.15) [14]. A series of chemical and physical methods have been developed for the synthesis of metal oxides at present, including electrospinning, hydrothermal method, thermal evaporation, etc. Among them, the hydrothermal method has advantages in both cost-effective large-scale production and precise control of product morphology and structure [28,29]. During hydrothermal processes, surfactants are usually introduced to alter the growth kinetics of metal oxides [30]. These surfactants include NaOH, glycerol, polyethylene glycol (PEG), sodium dodecyl sulfate (SDS), cetyltrimethylammonium bromide (CTAB), hexamethylenetetramine (HMTA), etc. [31]. The surfactant-assisted hydrothermal method has been proven to be an effective strategy for the controllable synthesis of various morphologies and structures. Nevertheless, pure ZnO-based gas sensors cannot be further applied in practical application due to their limitations, such as high operating temperature (300–500 °C) and poor selectivity. Therefore, it is urgent to improve their performance through noble metal modification, metal doping, and other methods [15,32].

Au, Ag, Pd, and Pt are typical noble metals that have been widely used as additives to enhance sensing performance, profiting from the effects of electronic and chemical sensitization on the surface electronic structure and configuration [33]. An improvement in sensitivity and reduced operating temperature and response/recovery time were achieved after doping noble metals. For instance, Barbosa et al. [34] modified SnO_2_ with Pt and Pd, respectively, and studied the relationship between sensitivity and operating temperature, as well as the concentration of the target gas. The results showed that both Pt and Pd modified SnO_2_ and exhibited an enhanced response and selectivity to H_2_. Compared with single metal nanoparticles, bimetallic alloy particles typically show stronger catalytic activity because of synergistic effects. For example, Liu et al. [35] designed and prepared AuPt bimetallic particle-modified ZnO nanowires on MEMS for detecting H_2_S. The response of bimetallic-decorated ZNWs is four times that of pure ZnO nanowires and it is improved compared to single-metal-decorated ZNWs. Moreover, the sensor exhibits a satisfying performance on gas selectivity and stability. Ahemad et al. [36] synthesized an AgAu alloy-decorated ZnO core–shell structure to detect ethanol, which showed high selectivity and good thermal stability. In summary, the combination of a flower structure with a large specific surface area and bimetallic decoration will provide a reliable strategy for further effectively improving the performance of ZnO sensors.

Herein, we prepared ZnO nanoflowers (ZNFs) through the hydrothermal method with glycerol as the surfactant, and decorated them with bimetallic AuPt. To absorb more oxygen molecules and sensing gases, the flower-like structure was designed to provide more active sites on its surface. Also, bimetallic modification reduces the activation energy of chemical reactions on the surface by altering the electronic configuration of ZnO so that more of the absorbed oxygen ions will react with toluene. Hence, the sensor exhibits high sensitivity to toluene and significantly reduces the optimal operating temperature. The response of AuPt@ZNFs reaches 69.7–50 ppm toluene with a short response time (22.4 s) at 175 °C, which is 9 times that of Au-decorated, 10 times that of Pt-decorated, and 30 times that of pure ZnO. The bimetallic AuPt@ZNFs also exhibits good selectivity. The strategy of combining a flower-like nanostructure and bimetallic decoration makes it effective for a toluene gas sensor to obtain better performance.

## 2. Results and Discussion

### 2.1. Morphology and Structure Properties

**Figure 1:**
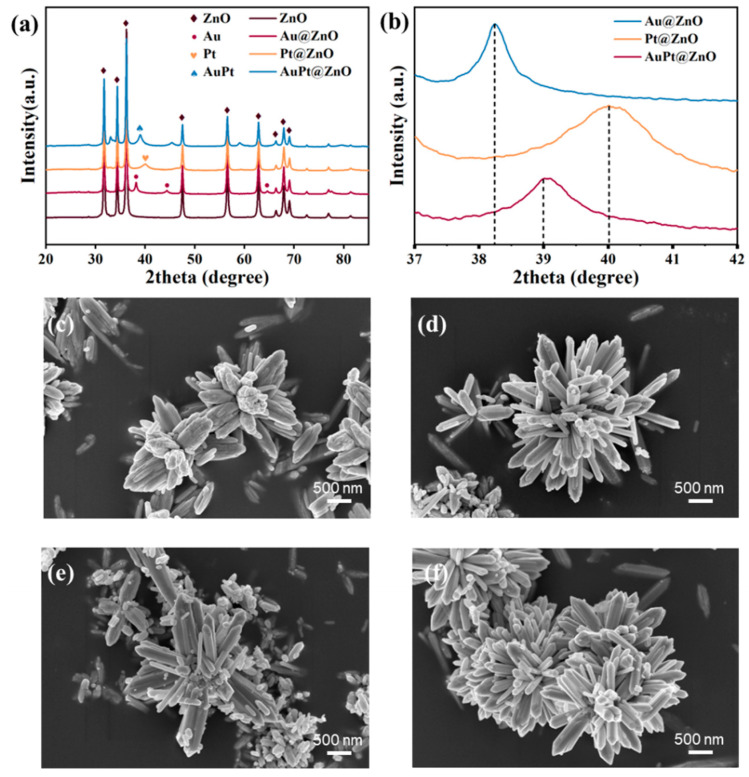
XRD results of samples (**a**) and selective region amplification of XRD for noble-metal-decorated ZnOs (**b**). SEM images of (**c**) ZnO nanoflowers, (**d**) Au@ZnOs, (**e**) Pt@ZnOs, and (**f**) AuPt@ZnOs.

The XRD patterns in Figure 1a show the phase structures of ZNF, Au@ZNF, Pt@ZNF, and AuPt@ZNF samples. The strong diffraction peaks observed in ZNFs perfectly match with the ZnOs (ICPDS: 36-1451). Compared to pure ZNFs, three new diffraction peaks in Au@ZNFs located at 38.18°, 44.36°, and 64.6° are consistent with the lattice planes of (111), (200), and (220) of FCC Au, respectively. The diffraction peak of Pt@ZNFs at 40.08° is a well-indexed characteristic peak, which perfectly matches with the Pt (111) planes. For the AuPt@ZNFs, a new diffraction peak that appeared at 39.02° was attributed to the (111) plane of the AuPt alloy (Figure 1b) since it was located between the (111) plane of Au and Pt. Briefly, XRD results indicate that both the single noble material and bimetallic alloy are successfully modified on ZnO nanoflowers.

Figure 1 shows microstructure characteristics of the prepared ZnO nanoflowers and ZNFs decorated with Au, Pt, and AuPt using SEM. As can be seen from the SEM images, all the samples exhibit flower-like nanostructures that are assembled by ZnO nanorods, causing an increase in the number of active sites on the surface during the target gas’s adsorption process and further enhancing performance. Au, Pt, and AuPt are decorated on ZnO successfully since nanoparticles are observed to be distributed on ZnOs’ surface in Figure 1d–f.

**Figure 2:**
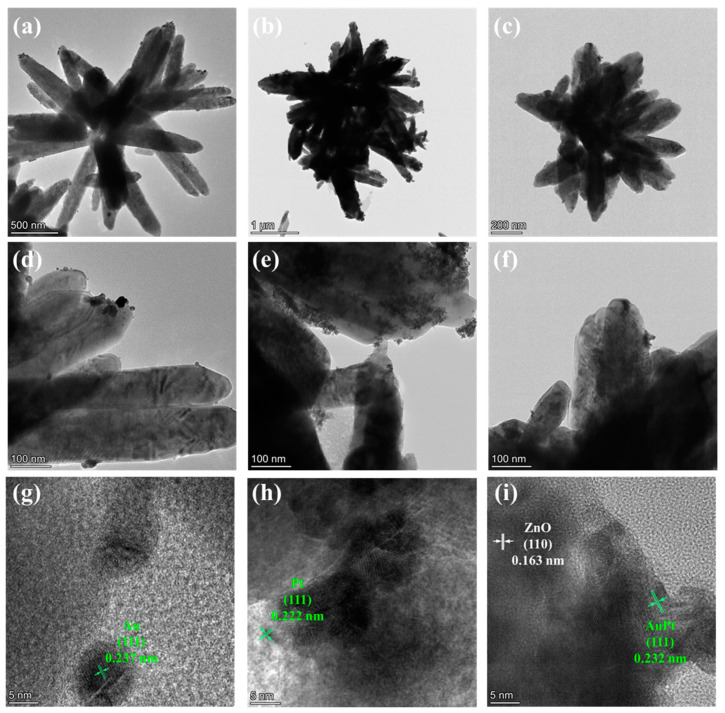
Images of TEM and high-resolution TEM (HRTEM): (**a**,**d**,**g**) Au@ZnO, (**b**,**e**,**h**) Pt@ZnO, and (**c**,**f**,**i**) AuPt@ZnO.

To further demonstrate the microstructure and morphology of the samples, TEM is used. As shown in the TEM images (Figure 2), the synthesized ZnO nanoflowers have a diameter of about 500 nm to 1 μm. For Au@ZNFs, the monometallic Au nanoparticles with a diameter of about 10 nm are decorated on the nanoflowers (Figure 2a,d,g). Pt nanoparticles decorated on ZnOs have a smaller diameter than Au and are densely aggregated (Figure 2b,e,h). The reason for the aggregation is the nanometer effect of Pt nanoparticles with high surface energy [37]. From the HRTEM images, the interplanar spacings of 0.237 nm that corresponds to the Au nanoparticles in Figure 2g and of 0.222 nm that corresponds to the Pt nanoparticles in Figure 2h are observed, further indicating the successful decoration of monometallic Au and Pt on the ZNFs. For AuPt@ZNFs (Figure 2c,f,i), the decorated nanoparticles have an interplanar spacing of 0.232 nm, which is between that of the Au (111) and Pt (111) planes (Figure 2i), indicating the formation of bimetallic AuPt and its successful decoration on ZnO nanoflowers. Moreover, this confirms the previous XRD results. The interplanar spacing of ZnO is 0.163 nm (Figure 2i), which matches the (110) plane of the ZnO wurtzite structure.

**Figure 3:**
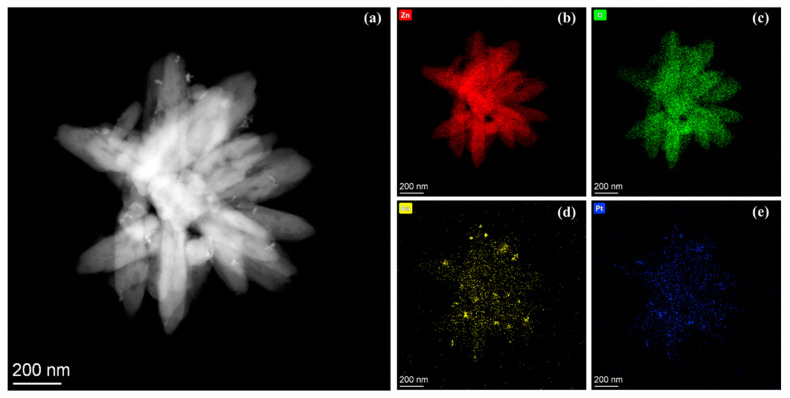
(**a**) HAADF-STEM image. Elemental images of AuPt@ZnO: (**b**) Zn; (**c**) O; (**d**) Au; (**e**) Pt.

As seen from the HADDF-STEM image of the AuPt@ZnO in Figure 3a, bimetallic AuPt nanoparticles are successfully decorated on the ZnO nanoflowers. Figure 3b–e exhibit the EDS mapping results of various elements in the samples, including Zn, O, Au, and Pt. The elemental mapping results further confirm that the AuPt alloys are successfully distributed on the ZnO nanoflowers.

**Figure 4:**
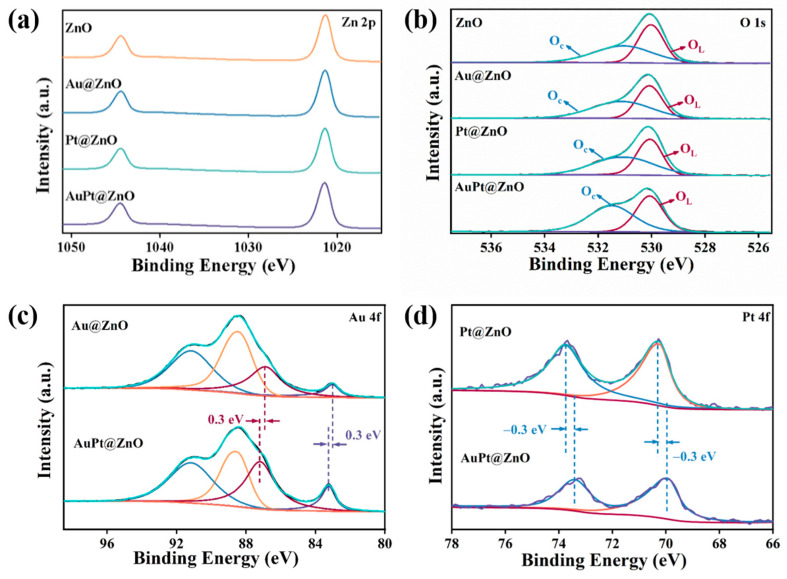
XPS spectra results: (**a**) Zn 2p and (**b**) O 1s for ZnO, Au@ZnO, Pt@ZnO, and AuPt@ZnO. Note that the blue curve, red curve and green curve correspond to O_c_ peak, O_L_ peak and fitted curve, respectively. (**c**) Au 4f for Au@ZnO and AuPt@ZnO. Note that the blue curve and orange curve correspond to Zn 3p. The red curve, purple curve and green curve correspond to Au 4f_5/2_, Au 4f_7/2_ and fitted curve, respectively. (**d**) Pt 4f for Pt@ZnO and AuPt@ZnO. Note that the blue curve, orange curve and green curve correspond to Pt 4f_7/2_, Pt 4f_5/2_ and fitted curve, respectively.

To illustrate the elemental valance states and surface compositions, XPS is applied for the qualitative and quantitative analyses of ZNFs, Au@ZNFs, Pt@ZNFs, and AuPt@ZNFs. The two peaks located at 1044.4 and 1021.3 eV of Zn 2p correspond to the ZnOs in the four samples (Figure 4a). After the decoration of the noble metals Au and Pt, the binding energy of the two Zn 2p peaks shifts to a high value because of the transfer of electrons from the ZnOs to monometallic and bimetallic nanoparticles, which makes samples decorated with metal nanoparticles more favorable to absorb oxygen from air. The two peaks of O 1s for the four samples are located around 531.1 and 530.1 eV (Figure 4b), and correspond to chemically adsorbed oxygen (O_C_) and lattice oxygen (O_L_), respectively [35]. To figure out the change in the proportion of adsorbed oxygen after decoration, the proportion of oxygen species in each sample is obtained with XPS calculations. Relative results are shown in Table 1. After the decoration of noble metals, the specific proportion of adsorbed oxygen increases, while AuPt@ZnO has the highest proportion (65.81%), which means it has the strongest capability for the adsorption of oxygen, higher than that of ZnO (54.92%), Au@ZnO (61.09%), and Pt@ZnO (59.19%).

The XPS curves in Figure 4c show the valence state of Au in both Au@ZNFs and AuPt@ZNFs. For the Au@ZNFs, peaks at 82.98 eV and 86.88 eV fit well with Au 4f_7/2_ and Au 4f_5/2_, respectively [37], while the other two peaks located at around 91.2 eV and 88.7 eV correspond to Zn 3p [38]. For the Pt@ZNFs in Figure 4d, Pt 4f_7/2_ and Pt 4f_5/2_ correspond to the peaks at 70.28 eV and 73.78 eV, respectively. Compared with bulk Au (84 eV) and Pt (71.2 eV), Au 4f_7/2_ and Pt 4f_7/2_ have a higher binding energy for Au@ZNFs, Pt@ZNFs, and AuPt@ZNFs, indicating the redistribution of electrons between ZnO and noble metals to achieve equilibrium at the Fermi level. Compared with the two peaks in Au@ZNFs, a positive shift of 0.3 eV in the binding energy of Au 4f_7/2_ and Au 4f_5/2_ is observed after the formation of the AuPt alloy. However, for Pt 4f_7/2_ and Pt 4f_5/2_ of the AuPt@ZNFs located at 73.48 eV and 69.98 eV, a negative shift of 0.3 eV occurs compared to that of the Pt@ZNFs. The opposite shift of increasing binding energy for Au 4f and decreasing binding energy for Pt 4f indicates the movement of electrons in AuPt from Au that have a lower work function of 5.1 eV in comparison to Pt, which have a higher work function of 5.6 eV [39,40], suggesting that electron redistribution occurs in the bimetallic AuPt alloy.

### 2.2. Performance of the Gas Sensors

**Figure 5:**
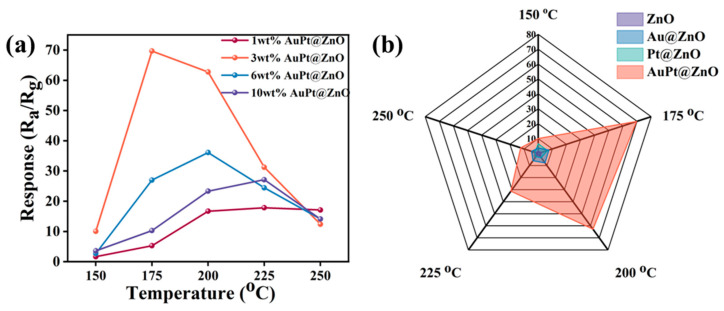
The sensing performance of ZnO-, Au@ZnO-, Pt@ZnO-, and AuPt@ZnO-based gas sensors: (**a**) Responses of AuPt@ZnO with different AuPt loadings. (**b**) Radar maps of responses at different operating temperatures.

A JF02F system is utilized for the performance characterization of ZNF-, Au@ZNF-, Pt@ZNF-, and AuPt@ZNF-based gas sensors. The content of decoration nanoparticles has a significant impact on sensing performance. The responses of ZnOs with different AuPt loadings of 1 wt%, 3 wt%, 6 wt%, and 10 wt% at various operating temperatures are investigated, as shown in Figure 5a. For a fixed content of AuPt-decorated ZnO nanoflowers, the response will increase to the maximum value at an optimal operating temperature and then gradually decrease at a higher temperature. This is attributed to the co-effect of kinetics and the equilibrium of adsorption and desorption [41]. At a low temperature, adsorbed oxygen will consume more free electrons to form chemically adsorbed oxygen ions as the temperature increases, which can cause the response value to increase. However, as the operating temperature continues to rise, the desorption rate of oxygen will exceed its adsorption rate, resulting in a decreased consumption of free electrons, leading to a reduced response value. For sensors at the same operating temperature, the response values will firstly increase and then decrease with the increasing content of AuPt. This is because when ZnO is decorated with a low content of noble metals—of which the electronic and chemical sensitization play a dominant role in regulating resistance—the Schottky barrier between the ZnO and noble metal nanoparticles increases with increasing loading content, leading to an increased sensor response. However, with a further increase in AuPt loading content, noble metal nanoparticles may occupy the active sites on the surface and undergo agglomeration on the ZnO’s surface at the same time [42], which will cause a decrement in the response value. Due to the above reasons, ZnO nanoflowers with 3 wt% AuPt loadings have a higher response to 50 ppm toluene than the other sensors at 150 °C, 175 °C, 200 °C, and 225 °C, and also have the highest response value of 69.7 at a lower optimal operating temperature of 175 °C. Therefore, the following investigation of gas sensing properties for AuPt@ZnO-based sensors is conducted at the fixed concentration of 3 wt%.

Obviously, the operating temperature has a significant impact on sensing properties. The optimal operating temperature of ZnO loaded with 3 wt% AuPt is 175 °C with exposure to 50 ppm toluene (Figure 5b). Moreover, AuPt@ZnO-based gas sensors exhibit the highest response compared to sensors that are based on other as-prepared sensing materials at all temperatures, which means that ZnO decorated with a bimetallic AuPt alloy is highly effective for sensing toluene.

**Figure 6:**
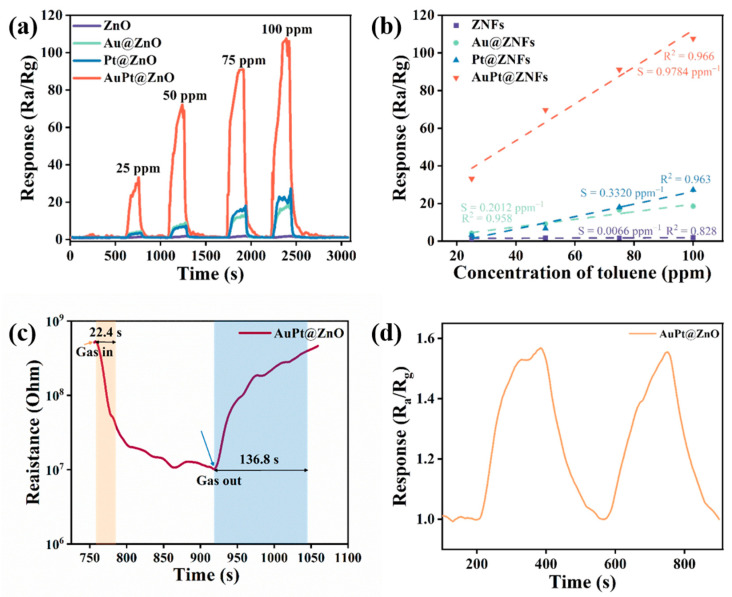
(**a**) Response curves of four samples at 175 °C with toluene at concentrations ranging from 25 to 100 ppm. (**b**) Curves showing the calculation of sensitivity towards toluene at 175 °C. (**c**) Response/recovery time of AuPt@ZnO at 175 °C with 50 ppm toluene. (**d**) Response of AuPt@ZnO-based sensor at 175 °C towards toluene at the limit of detection of 500 ppb.

The response curves of the four sensors at optimal working temperature are explored under different concentrations, ranging from 25 to 100 ppm of toluene (Figure 6a). It can be concluded from the curves that, as toluene concentration increases, the response of all sensing materials prepared also increases. Meanwhile, AuPt@ZNF-based sensors exhibit a response of 69.7 to 50 ppm toluene, which is the highest among all of the sensors, and is 30 times, 9 times, and 10 times higher than that of the ZNF- (2.2), Au@ZNF- (7.6), and Pt@ZNF (6.6)-based sensors, respectively. The responses of the four samples indicate that the bimetallic AuPt alloy enhances gas-sensing performance considerably compared to mono-metal nanoparticles, owing to the synergistic effect of bimetallic AuPt, as well as due to electronic and chemical sensitization. The responses of the sensors exhibit a linear relationship versus concentrations in the range of 25 to 100 ppm of toluene, as shown in Figure 6b. Among them, AuPt@ZnO-based sensors show an R^2^ of 0.966, which is close to 1 and higher than that of other sensors. This means that AuPt@ZnO-based sensors have a better linear relationship between the concentrations and responses, that is to say, the sensor is more suitable for toluene detection in working conditions. The slope of each fitting curve reflected the sensitivity of the sensors. A higher slope represents a higher sensitivity. Notably, AuPt@ZnO-based sensors depicted a sensitivity of 0.9784 ppm^−1^, which is higher than that of other sensors. The response/recovery time of bimetallic AuPt@ZNFs material is further investigated from the measured curves in Figure 6c. The sensor has a response time of 22.4 s when toluene gas is added and a recovery time of 136.8 s when the gas is excluded. The AuPt@ZnO-based sensor has a limit of detection (LoD) of 500 ppb, and the response at this concentration is 1.57 (Figure 6d).

**Figure 7:**
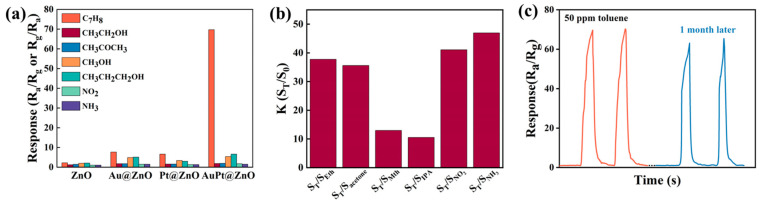
(**a**) Selectivity of samples at 175 °C facing various gases (50 ppm). (**b**) Cross-selectivity plot of toluene over other interfering gases at 175 °C. Note that Eth, Mth, and IPA correspond to ethanol, methanol, and isopropanol, respectively. (**c**) Long-term stability of AuPt@ZnO-based gas sensor.

Selectivity, which is generally obtained by comparing the response values of sensors to different gases with the same concentration at the same operating temperature, reflects the ability of sensors to accurately detect a target gas in the presence of interference gases. To investigate selectivity, all the prepared sensors were exposed to various interfering gases, including toluene, ethanol, acetone, methanol, isopropanol, NO_2_, and NH_3_ at their optimum working temperature. The results show that all sensors exhibited the highest responses to toluene among all of the tested gases (Figure 7a). Among them, sensors based on AuPt@ZNFs showed the highest selectivity, since their responses regarding toluene sensing were 10 times greater than those of other interfering gases, making them a good candidate for practical toluene sensing. Their good selectivity may be due to the high catalytic performance of bimetallic-decorated sensing material for toluene, which releases more electrons after a reaction of toluene molecules, resulting in greater resistance changes.

The cross-selectivity plot of the AuPt@ZnO-based sensor measured at 175 °C towards toluene with interfering gases like ethanol (S_T_/S_Eth_), acetone (S_T_/S_acetone_), methanol (S_T_/S_Mth_), isopropanol (S_T_/S_IPA_), NO_2_ (S_T_/$S_{{NO}_{2}}$), and NH_3_ (S_T_/$S_{{NH}_{3}}$) is shown in Figure 7b. The sensor has a higher cross-sensitivity and can be more easily interfered with by interfering gases if the ratio (K) is lower than 2. Thus, the AuPt@ZnO-based gas sensor exhibits less interference and cross sensitivity towards the tested gases since the ratio of all the gases is higher than 2. Thus, the sensor has a superior selectivity towards toluene in the presence of potential interfering gases [43].

The long-term stability and repeatability of the AuPt@ZNF-based gas sensor was further investigated with a response evolution method (Figure 7c) by retesting the response of the same sensor with the same measurement method after a month. The sensor was tested with two exposures to 50 ppm toluene, and the response along with the recovery capability were maintained with no decay. After another month, the same sensor was retested at 175 °C again with two exposures to 50 ppm toluene. The measured responses of the two exposures remain stable and still constitute to approximately 94% of the original response, proving good long-term stability and repeatability of the sensor.

The reproducibility of the AuPt@ZnO-based sensor was also investigated by reproducing the sensor through the same synthesis method of sensing material and using a fabrication method. The newly prepared AuPt@ZnO-based sensor exhibited comparable responses to the previous sensor towards different toluene concentrations at 175 °C (Figure S1, Table S1). Moreover, the newly prepared sensor also showed a good linear relationship between the response and toluene concentration (Figure S2), with an R^2^ of 0.990 and a sensitivity of 0.8499 ppm^−1^. Therefore, it is confirmed that the sensor has good reproducibility. Compared to other ZnO-based gas sensors that were investigated with regard to toluene detection, our sensor exhibited a relatively low working temperature and a higher response value, as shown in Table 2.

### 2.3. Performance of the Gas Sensors

**Figure 8:**
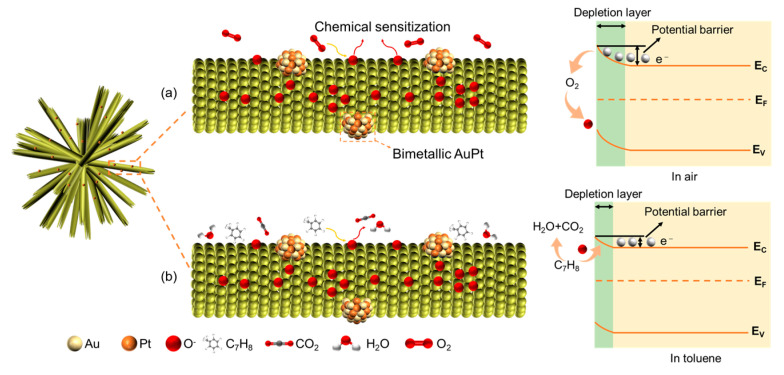
Schematic illustrations of sensing mechanism of AuPt@ZNFs (**a**) in air and (**b**) in toluene gas.

To explain the working mechanism of toluene sensors, both the band-bending theory and model for space charge was considered, as shown in Figure 8. When contacting air (Figure 8a), transfer of the captured electrons from the conduction band in ZnO to the adsorbed oxygen molecules occurs, forming a space charge layer, also known as the charge depletion layer. The width of the depletion layer is closely related to conductivity. The thicker the depletion layer, the greater the resistance of the ZnO [51], owing to the narrowing of the conduction band and the decrease in the electron concentration [14]. ZnO reacts with oxygen molecules in the air as follows:

(1) $$O_{2}(gas)↔O_{2}\left(ads\right)$$

(2) $$O_{2}\left(ads\right)+e^{-}↔O_{2}^{-}\left(ads\right)$$

(3) $$O_{2}^{-}\left(ads\right)+e^{-}↔2O^{-}(ads)$$

In the equation above, oxygen species include physically adsorbed O_2_(ads) and chemically adsorbed ions, that is, $O_{2}^{-}\left(ads\right)$ and $O^{-}(ads)$. The chemical adsorption of oxygen species is an energy activation process. The activation energy required for various oxygen species is different. At temperatures below 300 °C, $O^{-}(ads)$ is the main species [14].

When introducing a target gas (Figure 8b), the adsorbed oxygen ions are reduced by reacting with toluene. The release of electrons returning to the conduction band in the ZnO results in the contraction of the charge depletion layer and a decrease in resistance values. The relative reaction equation is as follows:

(4) $$C_{7}H_{8}+18O^{-}\left(ads\right)↔7{CO}_{2}+4H_{2}O+18e^{-}$$

A higher response to toluene of the AuPt@ZNF-based sensor is caused by more $O^{-}\left(ads\right)$ reacting with the target gas and releasing a quantity of electrons, which corresponds to the XPS results described earlier.

Compared to the original ZnO-based sensor, the sensing performance is improved in the AuPt@ZnO-based sensor, which is the co-effect of (i) the electronic and chemical sensitization of noble metals; (ii) the synergistic effect of bimetallic AuPt; (iii) the interaction between noble metals and the −CH_3_ group of toluene; (iv) the electron release theory [52].

Firstly, the bimetallic AuPt alloy has a work function in the range of 5.1~5.6 eV, which is higher than ZnO (4.7 eV). The difference in the work function can lead to a depletion layer forming at the contact interface of the ZnO and AuPt, and a transfer of electrons from ZnO to bimetallic AuPt to reach an equilibrium in relation to the Fermi level. During the process, a Schottky barrier is formed, causing a thicker depletion layer, which will not only prevent the recombination of separated electron–hole pairs, but also causes more chemically adsorbed oxygen ions to react with the target gas of toluene, and thus more released electrons, leading to a jump in resistance and improving the sensor’s response [35,53]. On the other hand, bimetallic AuPt promotes the dissociation of oxygen molecules, generating a high concentration of chemically adsorbed oxygen ions due to the spillover effect and its catalytic properties. These oxygen ions overflow to the grain surface and allow more toluene molecules to participate in the reaction on ZnO’s surface and improve the response [54,55]. Secondly, loaded AuPt nanoparticles can impact the electron configuration of ZnO’s surface through a synergistic effect. Moreover, the activation energy needed for a chemical reaction can also be reduced by the synergistic catalysis of Au and Pt, accelerating the speed of the reaction between the target gas and the chemically adsorbed ions, and shortening the response and recovery time. Thirdly, ZnO tends to interact with π-conjugated benzene rings in toluene molecules and forms π−π stacking. π electrons, of which the density will increase due to the influence of a methyl group in toluene, will reconstruct the surface of charge redistribution by interacting with the 2p orbital of surface oxygen in the ZnO and cause physical adsorption [56]. For ZnO decorated with noble metals, to figure out the effect of the −CH_3_ group and noble metals, both electronic and steric effects should be taken into consideration. From the perspective of the steric effect, the adsorption of toluene will become difficult because of the repulsive effect between the methyl group and the surface of a noble metal. However, from the perspective of the electronic effect, a reduced potential barrier of toluene adsorption occurs due to the interaction between the πCH_3_ level and the Fermi level; on the one hand, electrons are more likely to transfer from the πCH3 level to the Fermi level. However, the Fermi level also has a reverse contribution to the πCH_3_ level. Compared to the steric effect, the electronic effect plays a dominant role in the adsorption process, thus reaching the purpose of improving the response of a toluene sensor [53]. In addition, the increasing response is also related to the lower dehydrogenation enthalpy change of toluene [57].

Finally, the high selectivity of sensors towards toluene can be explained by the electron release theory and bond dissociation energy. On the one hand, in contrast to the other gases, the reaction between ZnO and toluene will release more electrons while the concentration of target gas is being determined, thus reaching a higher response in a short time [58,59,60,61]. On the other hand, the low bond dissociation energy of toluene has a positive impact on improving selectivity. The change of the standard enthalpy generated when a chemical bond dissociates at 298 K is called bond dissociation energy. Smaller energy required for bond dissociation means that the chemical bonds are more likely to break, resulting in more chemical reactions during the adsorption process of toluene. From Table 3 below, it can be seen that toluene has a lower energy for bond dissociation.

## 3. Materials and Methods

### 3.1. Preparation of ZNFs and ZNFs Decorated with Au, Pt, and AuPt Alloy

A mixed solution of 7 mL glycerol (Sinopharm Chemical Reagent Co., Ltd., Shanghai, China), 7 mL absolute ethanol (Shanghai Titan Technology Co., Ltd., Shanghai, China), and 10 mL deionized water (DIW) was stirred and for 1 h. The addition of 0.07 mM Zn(NO_3_)_2_·6H_2_O (Sinopharm Chemical Reagent Co., Ltd.) and 0.8 mM NaOH (AR, Shanghai Aladdin Biochemical Technology Co., Ltd., Shanghai, China) was carried out before 1 h of stirring for sufficient mixing of the reactants. A 50 mL Teflon-lined stainless steel autoclave, which maintained the mixed solution at 120 °C for 12 h, was used for hydrothermal reactions. The products were obtained by centrifugating at 8000 rpm for 5 min, and they were several times with DIW and absolute ethanol. Afterwards, white ZNFs powders were obtained by drying the products overnight at 80 °C.

For the decoration of Au and Pt nanoparticles on ZNFs, 470 μL of both 20 mM HAuCl_4_ (Beijing Innokai Technology Co., Ltd., Beijing, China) and H_2_PtCl_6_ (Shanghai Aladdin Biochemical Technology Co., Ltd.) were added to 18 mL of a uniformly dispersed solution of 60 mg ZNFs to obtain ZNFs decorated with Au and Pt, respectively. Then, the solution was stirring for 0.5 h. After that, the addition of an appropriate amount of 100 mM freshly prepared NaBH_4_ (Sinopharm Chemical Reagent Co., Ltd.) was carried out before fully stirring for 3 h to completely reduce Au and Pt. Finally, Au@ZNF and Pt@ZNF powders were successfully obtained via centrifugation and drying.

To obtain AuPt@ZNFs, 235 μL of 20 mM HAuCl_4_ and 235 μL of 20 mM H_2_PtCl_6_ were mixed into ZNF suspensions, with the other steps being the same as for the synthesis of Au@ZNFs and Pt@ZNFs.

### 3.2. Characterization of Morphology and Structure

Observation of morphology was achieved with scanning electron microscopy (SEM, RISE-MAGNA, TESCAN, Brno, Czech Republic). The analysis of element composition and features for the synthesized samples was implemented via transmission scanning electron microscopy (TEM, TALOS F200X G2, Thermo Scientific, Waltham, MA, USA) and high-resolution TEM (HRTEM, Thermo Scientific, USA). The component and crystalline phase of samples was characterized by utilizing X-ray diffraction (XRD, D8 ADVANCE DA Vinci, Bruker, Germany) with Cu Kα radiation source. To obtain element and valence state information about the surface of samples, X-ray photoelectron spectroscopy (XPS, AXIS UltraDLD, Shimadzu, China) was utilized.

### 3.3. Fabrication and Measurements of Gas Sensors

**Figure 9:**
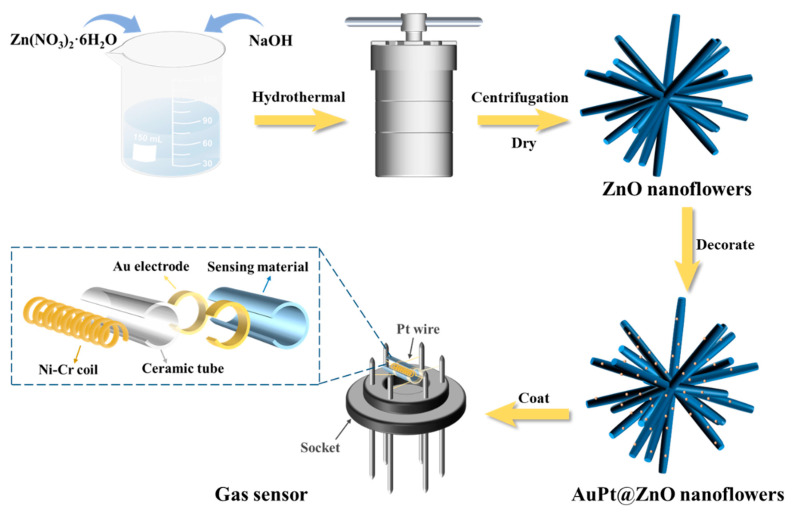
Fabrication of gas sensor based on AuPt@ZnO nanoflowers.

The preparation process of the sensors is shown in Figure 9. In total, 10.0 mg of the synthesized products and 0.5 mL ethanol were mixed and then uniformly coated on the alumina ceramic tube with a small brush and dried overnight. The sensors were prepared by welding ceramic tubes on hexapod sockets. The operating temperature, which has a specific relationship with heating voltage, was adjusted with a Ni-Cr alloy coil that passed through the tube and was welded onto the socket. To maintain a good stability for further measurement, the sensors needed to be aged at 175 °C for 24 h. Then, sensing performance of the sensors was measured via the gas sensing test system (JF02F, Guiyan Jinfeng Technology Co., Ltd., China). C_6_H_5_CH_3_ (toluene), CH_3_COCH_3_, CH_3_CH_2_OH, CH_3_CH_2_CH_2_OH, CH_3_COOH, NH_3_, and NO_2_ were listed as target gases, of which specific concentrations in dry air were precisely regulated by a gas distribution control box. For reducing gases, a response was defined as follows:

R_s_ = R_a_/R_g_ (5)

For oxidizing gases, a response was defined as

R_s_ = R_g_/R_a_ (6)

Sensors exposed to air and target gas had a resistance of R_a_ and R_g_, respectively. The time required for a 90% change of resistance from making contact with the target gas to reaching adsorption equilibrium was response time, while time required for a 90% change of resistance from the detachment of the target gas to reaching desorption equilibrium was recovery time.

## 4. Conclusions

In this work, ZnO nanoflowers were successfully synthesized through the hydrothermal method while being decorated with noble metals. Owing to the unique nanoflower-like structure of ZnO and its interaction with the -CH_3_ group of toluene, active sites on the surface required for toluene adsorption were provided in large quantities. An improvement in the sensing performance of the AuPt@ZNF-based sensor was the result of a combination of electronic sensitization, the spillover effect, the synergistic effects of the AuPt bimetal, and the unique electronic effects between noble metals and toluene. The high selectivity of the sensor towards toluene can be explained by the electron release theory and bond dissociation energy. The AuPt@ZNF-based sensor achieved an improvement in its response of 69.7 towards 50 ppm toluene at 175 ° C and obtained satisfactory stability. In summary, the decoration of AuPt bimetals provides a feasible approach for the practical application of toluene sensors with satisfactory performance.

## Figures and Tables

**Table 1:** The proportion of oxygen species in the four samples calculated via XPS.

Samples	O_c_ (%)	O_L_ (%)
ZnO	54.92	45.08
Au@ZnO	61.09	38.91
Pt@ZnO	59.19	40.81
AuPt@ZnO	65.81	34.19

**Table 2:** Performance comparison of ZnO-based sensors for toluene detection.

Materials	Morphology	Concentration (ppm)	Response (Ra/Rg)	Operating Temperature (°C)	Response/Recovery Time (s)	Reference
Au-ZnO	Nanowire	100	8.623	340	60/180	[44]
Pt-coated SnO_2_-ZnO	Core–shell nanofibers	50	3.14 (Voltage 20 V)	RT	-/-	[45]
ZnO/ZnFe_2_O_4_	Spherical urchin-like core–shell structure	100	59.41	275	3/189	[1]
ZnO@Co_3_O_4_	Hollow cubes	100	26.4	290	11.2/12.5	[46]
Pt@ZnO-TiO_2_	Nanotube	5	25	300	7.5/20.1	[47]
ZnO/graphene	Nanoparticle	1	12.57	300	-/-	[48]
In_2_O_3_-ZnO	Nanofiber	100	14.63	RT	14/201	[49]
NiO/ZnO	Nanoflower	95	19.1	300	70/55	[50]
AuPt@ZnO	Nanoflower	50	69.7	175	22.4/136.8	This work

**Table 3:** Energy required for bond dissociation of various gases [62,63,64].

Gas	Toluene	Acetone	Ammonia	Methanol	Ethanol	Isopropanol	Nitrogen Dioxide
Bond	CH_3_-C_6_H_5_	H-CH_2_COCH_3_	H-NH_2_	H-CH_3_O	H-CH_3_CH_2_O	H-CH_3_CH_2_CH_2_O	O-NO
Bond dissociation energy (kJ/mol)	389	393	435	438	438	442	305

## Data Availability

The data presented in this study are available upon request from the authors.

## Supplementary Materials

The following supporting information can be downloaded at: https://www.mdpi.com/article/10.3390/molecules29071657/s1, Figure S1: Response curve of reproduced AuPt@ZnO-based sensor at 175 °C towards toluene at the concentration ranging from 10 to 200 ppm; Figure S2: Fit curves of the response of the reproduced AuPt@ZnO-based sensor versus toluene concentration at 175 °C; Figure S3: Hysteresis characteristics of the AuPt@ZnO-based sensor; Table S1: The comparison of responses at different concentration of toluene between the newly prepared and previous sensor at 175 °C.
