# Supplementary material for: Highly Sensitive and Selective Toluene Gas Sensors Based on ZnO Nanoflowers Decorated with Bimetallic AuPt
Source: Molecules. 2024 Apr 7;29(7):1657. doi: 10.3390/molecules29071657 (PMC11013457; doi:10.3390/molecules29071657)
Supplement: Supplementary file 1 [file molecules-29-01657-s001.zip › molecules-2931149-supplementary.pdf]

Supporting Information

Highly Sensitive and Selective Toluene Gas Sensors Based on ZnO Nanoflowers Decorated with Bimetallic AuPt

Huiting Peng [1], Yiping Liu [1], Yinfeng Shen [1], Ling Xu [2], Jicun Lu [2], Ming Li [1], Hong-Liang Lu [3,*] and Liming Gao [1,*]

1 State Key Laboratory of Metal Matrix Composites, School of Material Science and Engineering, Shanghai Jiao Tong University, Shanghai 200240, China

2 Guanghua Lingang Engineering Application Technology Research and Development (Shanghai) Co, Ltd., Shanghai 201306, China

3 State Key Laboratory of ASIC and System, Shanghai Institute of Intelligent Electronics & Systems, School of Microelectronics, Fudan University, Shanghai 200433, China

* *Correspondence: honglianglu@fudan.edu.cn (H.-L.L.); liming.gao@sjtu.edu.cn (L.G.)*

Table S1. The comparison of responses at different concentration of toluene between the newly prepared and previous sensor at 175 °C.

Response / Concentration (ppm) / Sensors	25	50	75	100
Previous sensor	33.2	69.7	91.1	107.6
Newly prepared sensor	41.3	66.8	88.1	107.0

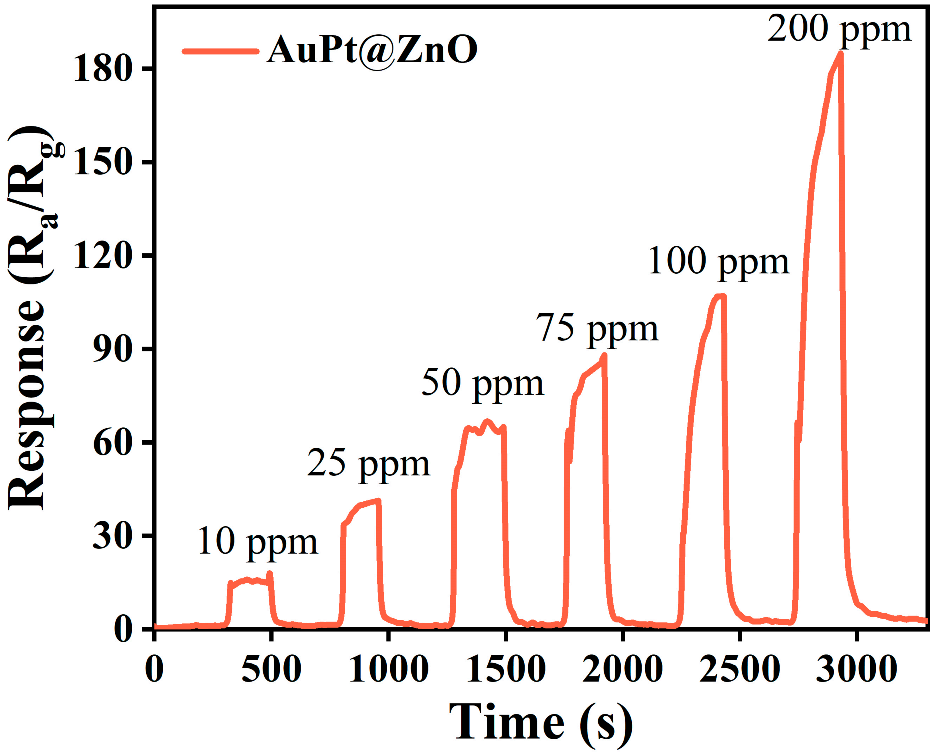

Figure S1. Response curve of reproduced AuPt@ZnO-based sensor at 175 °C towards toluene at the concentration ranging from 10 to 200 ppm.

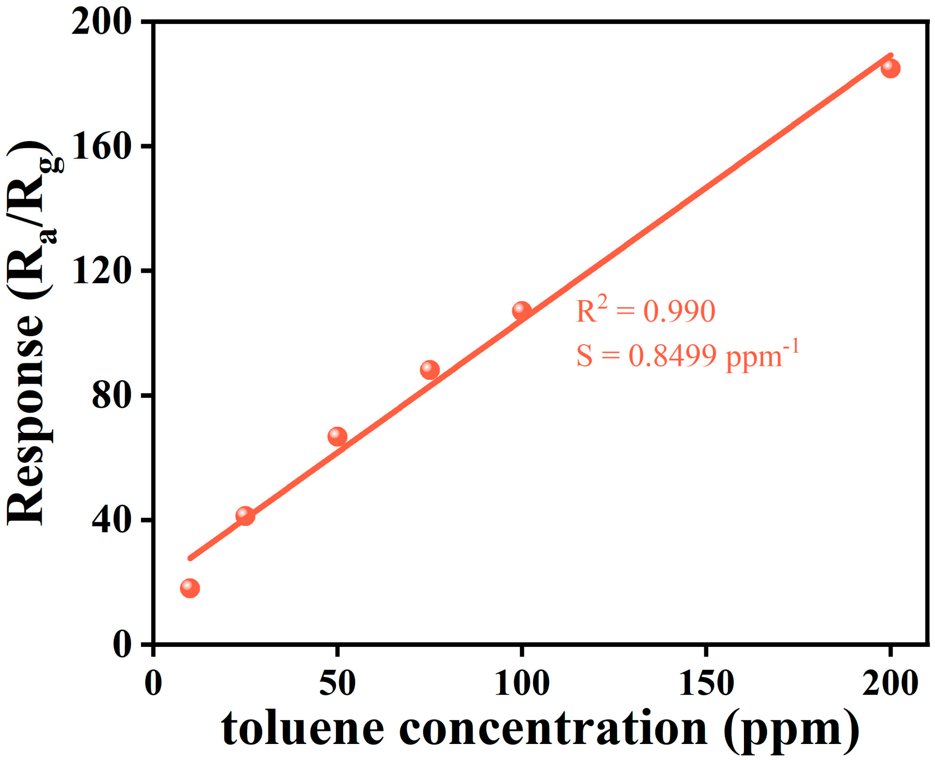

Figure S2. Fit curves of the response of the reproduced AuPt@ZnO-based sensor versus toluene concentration at 175 °C.

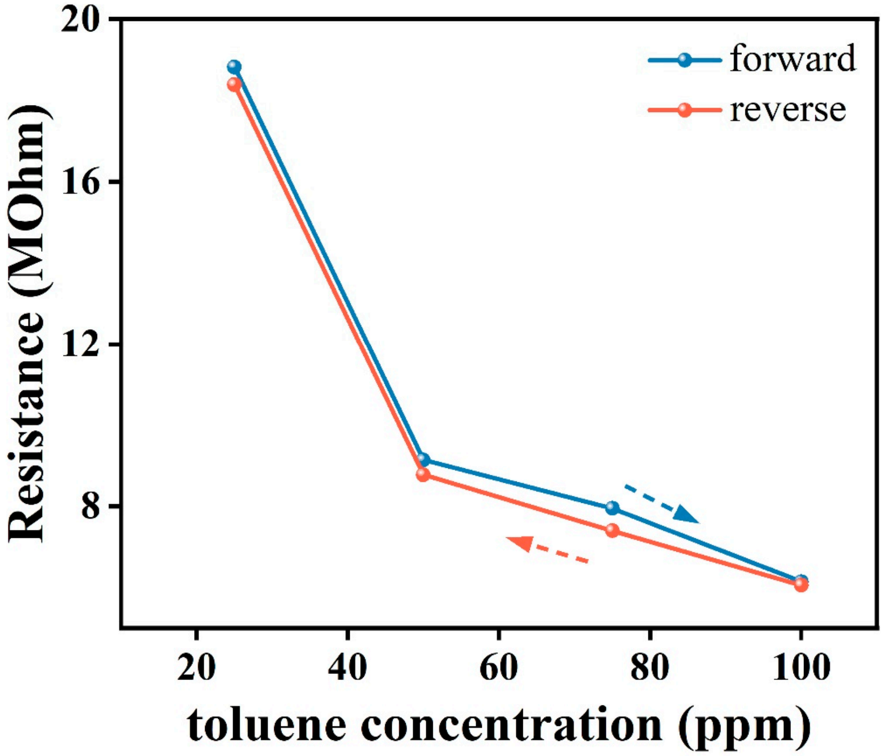

Figure S3. Hysteresis characteristics of the AuPt@ZnO-based sensor.
